# Prehabilitation in Adults Undergoing Cancer Surgery: A Comprehensive Review on Rationale, Methodology, and Measures of Effectiveness

**DOI:** 10.3390/curroncol31040162

**Published:** 2024-04-09

**Authors:** Carlos E. Guerra-Londono, Juan P. Cata, Katherine Nowak, Vijaya Gottumukkala

**Affiliations:** 1 Department of Anesthesiology, Pain Management & Perioperative Medicine, Henry Ford Health, Detroit, MI 48202, USA; cguerra1@hfhs.org (C.E.G.-L.); knowak2@hfhs.org (K.N.); 2 Department of Anesthesiology and Perioperative Medicine, The University of Texas MD Anderson Cancer Center, Houston, TX 77030, USA; jcata@mdanderson.org

**Keywords:** cancer, surgery, prehabilitation, preoperative, exercise, complications

## Abstract

Cancer surgery places a significant burden on a patients’ functional status and quality of life. In addition, cancer surgery is fraught with postoperative complications, themselves influenced by a patient’s functional status. Prehabilitation is a unimodal or multimodal strategy that aims to increase a patient’s functional capacity to reduce postoperative complications and improve postoperative recovery and quality of life. In most cases, it involves exercise, nutrition, and anxiety-reducing interventions. The impact of prehabilitation has been explored in several types of cancer surgery, most commonly colorectal and thoracic. Overall, the existing evidence suggests prehabilitation improves physiological outcomes (e.g., lean body mass, maximal oxygen consumption) as well as clinical outcomes (e.g., postoperative complications, quality of life). Notably, the benefit of prehabilitation is additional to that of enhanced recovery after surgery (ERAS) programs. While safe, prehabilitation programs require multidisciplinary coordination preoperatively. Despite the existence of numerous systematic reviews and meta-analyses, the certainty of evidence demonstrating the efficacy and safety of prehabilitation is low to moderate, principally due to significant methodological heterogeneity and small sample sizes. There is a need for more large-scale multicenter randomized controlled trials to draw strong clinical recommendations.

## 1. Introduction

Surgery is essential to improve survival in patients with solid malignancies. By 2040, the global demand for cancer surgery is estimated to increase by 52%, representing nearly 14 million cases [1]. Despite its benefits, surgery is known to negatively affect a patient’s quality of life and functional status [2]. The postoperative journey is also fraught with postoperative complications, which can be influenced by the type of surgery, medical history, and functional status [3]. Postoperative complications are a major concern in cancer patients because they are strongly associated with worse overall and/or disease-specific survival [4,5,6,7], even if the patients survive them [8]. Over the last two decades, there has been a growing interest in the implementation of preoperative interventions to improve a patient’s functional status. This is known as prehabilitation [9]. Prehabilitation programs are particularly appealing for cancer surgeries and represent the population of interest in nearly half of all randomized controlled trials to date [10].

The need for prehabilitation rests strongly on the association between preoperative physical fitness and perioperative outcomes [11]. By improving a patient’s functional status, prehabilitation aims to hasten recovery and ameliorate postoperative risks. Over time, prehabilitation has expanded to other dimensions that are thought to affect the postoperative course, directly or indirectly. In the continuum of perioperative management, prehabilitation must be differentiated from the treatment of specific conditions (e.g., anemia, smoking cessation, or depression) and enhanced recovery after surgery (ERAS). Consequently, working definitions stipulate that it must begin more than 7 days before the time of surgery and last for a minimum of 7 days [10].

Previous systematic and scoping reviews have noted the paucity of high-quality data supporting the benefits of prehabilitation in surgical patients [12,13,14,15]. Perhaps the biggest challenge in ascertaining its effectiveness is the large variability of interventions, schedules, durations, and modalities. More recently, several clinical trials evaluating prehabilitation in cancer surgery have been published [16,17,18]. This study follows the recommendations of the SANRA scale for narrative reviews [19]. Here, we aim to guide clinicians and researchers seeking to implement or study prehabilitation in the oncological surgical population. Thus, we performed a comprehensive narrative review behind the rationale, methodology, and measures of effectiveness of prehabilitation in patients undergoing cancer surgery. To provide a thorough assessment of the most recent publications, an electronic literature search was conducted by a librarian in Ovid Medline for English-language publications within the last 10 years using MeSH terms related to prehabilitation, preoperative exercise, surgical oncology, cancer, and surgical procedures, in addition to other valuable literature sources. Article selection was guided by expert knowledge and overall representativeness, such as prioritizing results from systematic reviews, meta-analyses and landmark clinical trials. 

The manuscript is structured around the following four questions:

- What is the rationale behind prehabilitation in cancer patients?
- What are the prehabilitation strategies used in cancer surgical patients?
- How is the effect of a prehabilitation strategy measured?
- What is the suggested effective duration of a prehabilitation cancer program?

## 2. What Is the Rationale of Prehabilitation in Cancer Patients?

Cancer is a heavy burden in multiple aspects of a survivor’s life, including their mental health [20,21], physical health [22], financial condition [23,24], and even their caregivers [25]. From a mental health perspective, cancer patients experience higher preoperative hospital and surgery-related stress [26]. In turn, preoperative mental health has been associated with postoperative complications. For instance, one study showed that preoperative composite scores of mental health were significantly lower in patients who experienced complications after radical cystectomy [27]. Likewise, a secondary analysis of the VISION (vascular events in noncardiac surgery patient cohort evaluation) study associated preoperative distress with greater odds of re-hospitalization and longer hospital stay, a finding that remained relevant in the subgroup of patients undergoing cancer surgery [28]. Preoperative anxiety is reported in at least one third of women undergoing breast cancer surgery [20]. Higher scores on patient-reported anxiety and catastrophizing predict higher persistent acute pain trajectories [29]. Furthermore, preoperative mental health predicts postoperative mental health [30]. Thus, strategies to reduce preoperative mental distress and anxiety are justified in their potential benefits on postoperative outcomes like complications, length of stay, and acute and chronic pain. 

Physical health is at the center of most prehabilitation interventions. Regardless of the cause, an overall decline in physical health can be evidenced by sarcopenia, malnutrition, and/or diminished cardiorespiratory fitness. Sarcopenia is characterized by decreased muscle strength and decreased muscle quantity or quality, with or without decreased physical performance [31]. It can be screened with self-report questionnaires (e.g., SARC-F) and confirmed with a combination of strength testing (i.e., grip strength or chair stand test) and measures of muscle quantity (e.g., computed tomography, bioelectric impedance). In older cancer patients, the prevalence of sarcopenia can range between 18.5 and 83% [32]. Furthermore, skeletal muscle mass decreases in most patients following neoadjuvant chemoradiotherapy for rectal adenocarcinoma, effectively increasing the prevalence of sarcopenia prior to surgery [33]. Finally, sarcopenia is known to predict worse outcomes in several types of cancer, including esophageal [34], ovarian [35], colorectal [36], and head and neck cancers [37].

Nutritional derangements are common in cancer patients and are also associated with poor outcomes. In a study of 637 patients, Zhang et al. showed that up to 43.7% of patients could be identified as being at a high risk of malnutrition [38]. Furthermore, the presence of malnutrition is associated with a higher proportion of postoperative complications (81% vs. 42%, *p* = 0.013) in adults undergoing pharyngectomy or laryngectomy procedures [39]. In a meta-analysis of 22 studies (*n* = 9332), the risk of malnutrition ranged from 12.8% to 80.8%. Overall, a high risk of malnutrition was associated with more than two times the odds of any postoperative complication (OR 2.27, 95% CI 1.81–2.84) and worse survival (HR 1.66, 95% CI 1.4–1.97) [40]. The high incidence of malnutrition in cancer patients is also attributed to inflammatory mediators released in response to tumor cells [41].

As of now, recommendations from the European Society for Clinical Nutrition and Metabolism (ESPEN) on perioperative nutrition have focused on the postoperative period as part of an enhanced recovery program [42]. Nonetheless, nutritional prehabilitation has been acknowledged as an area of focus for future updated guidelines [43]. Studies on nutritional prehabilitation have used a variety of measurements to assess baseline nutritional status and identify cancer patients who are most at-risk of malnutrition. These incorporate objective nutrition parameters (anthropometric, biochemical, and immunological), subjective measurements (weight change and dietary intake change), and physical assessments (loss of muscle and fat mass) [39,44,45,46]. It is important to note that, although they are separate conditions, sarcopenia and malnutrition often coexist [42].

**Figure 1:**
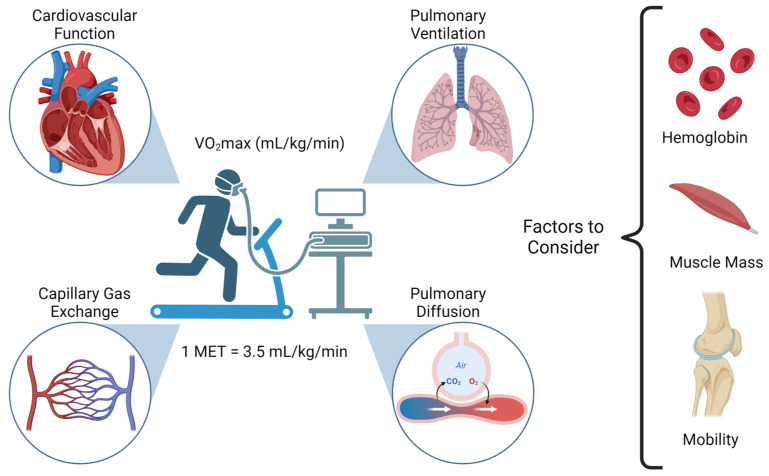
Assessment of cardiorespiratory fitness. Adequate cardiorespiratory fitness requires optimal interaction between the cardiovascular and respiratory systems to supply oxygen to and eliminate carbon dioxide from the tissues. Results from cardiopulmonary testing reflect adequacy of pulmonary ventilation and diffusion, cardiovascular function (e.g., systolic function), and capillary gas exchange. Cardiopulmonary exercise testing can also be affected by hemoglobin concentration, muscle mass, and inadequate mobility (e.g., arthritis).

Adequate cardiorespiratory fitness (CRF) requires the appropriate function of multiple body systems and reflects their collective ability to deliver oxygen to tissues and eliminate the carbon dioxide generated by them (Figure 1). CRF can be measured directly or indirectly and is often expressed as the maximal oxygen consumption (VO_2_max or peak VO_2_ for those with cardiovascular disease), also referred to as maximal aerobic capacity. Other measures of CRF include the anaerobic threshold and the ventilatory equivalents of oxygen and carbon dioxide [47]. In the general population, the evidence strongly supports a dose–response effect between CRF and health outcomes, where even a small increase in VO_2_max of 1–2 METs (Metabolic Equivalent Tasks, 1 MET = 3.5 mL/kg/min) is associated with a 10–30% decrease in cardiovascular events [48]. Perioperatively, this issue holds increasing relevance considering that cardiac events are a leading cause of early postoperative morbidity and mortality [49]. Pulmonary complications can also be predicted by decreased functional capacity. In adults undergoing major abdominal cancer surgeries, Rezaian et al. found that VO_2_max was significantly lower (11.9 vs. 16.8 mL/kg/min, *p* < 0.0001) in patients who developed postoperative respiratory failure. Alone, CRF had a low to moderate correlation with the ARISCAT pulmonary risk score [50]. In a recent meta-analysis, Steffens et al. found that a higher VO_2_max was strongly associated with improved outcomes in adults undergoing cancer surgery [51]. The studies included defined VO_2_max thresholds below which postoperative complications are more likely to occur, most of them ranging between 10 and 20 mL/kg/min.

From a patient-centered perspective, prehabilitation has shown promising findings [52]. In one study, Jandu et al. found that 81.5% of patients would join a prehabilitation program if available, and that 60% of them considered that prehabilitation helped them prepare mentally for surgery [53]. In addition, it can offer multiple qualitative benefits. In one study, Powell et al. conducted a series of interviews with clinicians and patients involved in a multimodal prehabilitation program for cancer surgery [54]. Patients reported better mood and the opportunity to shift focus away from cancer. Furthermore, they gained more sense of control over their cancer experience while also being supported in the process.

In summary, prehabilitation for cancer surgery is justified by the multiple effects that cancer has on a patient’s mental and physical health, and the strong association between those measures and postoperative outcomes. It has been found that patients with a CRF below the threshold of 10–20 mL/kg/min have an increased risk of postoperative complication. Furthermore, most surgeries for cancer are elective and many of them require neoadjuvant therapy, which allows for the time needed to implement a prehabilitation strategy. Prehabilitation provides clinicians and healthcare systems the opportunity to support and empower patients to gain control of their cancer experience while improving their health.

## 3. What Prehabilitation Strategies Are Used in Cancer Surgical Patients?

Prehabilitation can be unimodal or multimodal. Consistent with recent scoping reviews [10,55], four types of prehabilitation interventions have been described. 

### 3.1. Exercise

This is undoubtedly the most common intervention. Exercise can be in the form of aerobic training, resistance training, or both. Aerobic training includes high-intensity interval training [56,57] and/or moderate-intensity endurance training. Given the need for results over a short period of time, high-intensity interval training is generally preferred [58]. On average, exercise programs have a mean of 3.5 sessions per week. Each aerobic session consists of 10–15 min of warm-up, followed by 30 min of training with a wide range of heart rate goals [14]. In contrast, resistance training may only require 10–20 min [59]. Common resistance training includes resistance bands and free weights, where participants can perform sets of repetitions in one or more muscle groups with the goal of achieving a pre-specified rating of perceived exertion [60], as described by Borg et al. [61]. While aerobic training aims at improving VO_2_max (or its surrogates), the goal of resistance training is to increase muscle mass. Therefore, resistance training is crucial in addressing sarcopenia. 

For implementation, two major strategies have been used: center-based and home-based strategies [62]. Center-based strategies have the benefit of increased supervision and the ability to ensure adherence to the goals of the session. In turn, home-based strategies can be less expensive and more accommodating for patients’ schedules. Interestingly, patient preferences seem evenly divided between them [53]. 

### 3.2. Nutritional

Due to the variable nutritional needs among patients, a baseline nutritional assessment should be conducted to guide prehabilitation strategies. Various validated tools are available to assess nutritional status and malnutrition risk, including the Malnutrition Universal Screening Tool (MUST), Nutritional Risk Screening (NRS-2002), Patient-Generated Subjective Global Assessment (PG-SGA), Skeletal Muscle Index (SMI), albumin, the controlling nutritional status (CONUT) score, and Prognostic Nutritional Index (PNI). Ideally, a combination of these tools should be used to capture a comprehensive overview of the patient’s nutritional status [63]. Among the most useful measures are the patient’s BMI, weight loss, food intake, and preoperative laboratory values such as serum albumin. A preoperative serum albumin level of <4 g/dL is predictive of postoperative complications, and as serum albumin decreases, the risk of complications and mortality increases [64]. 

Nutritional support can include nutritional counseling and/or supplementation and typically focuses on meeting energy and protein requirements. Studies have used energy requirements between 25 and 30 kcal per kg of body weight, and 1.5 g per kg of body weight for protein support [16,59]. Immunonutrition (IMN) is a prehabilitation strategy that involves supplementation with selected amino acids and proteins. IMN is a targeted approach intended to modify the immune and inflammatory response, as well as modulate protein synthesis [65]. Two amino acids of interest for prehabilitation supplementation are arginine and glutamine, as these are critical in wound healing and immune response [66,67]. These are conditionally essential amino acids and must be obtained from exogenous sources, with a recommended daily allowance of 5–6 g of arginine and 1 g of glutamine [68].

It is important to note that exercise is important in nutritional efforts as it promotes the maintenance of or increase in muscle mass and reduces catabolism. As a result, exercise is strongly recommended in the ESPEN guidelines for cancer patients [69].

### 3.3. Psychosocial

Studies have also referred to this as medical coaching [60]. During the initial assessment, a discussion with the patient can help identify social support systems, emotional management, and recognition of strengths, among others. Specific psychological interventions include anxiety reduction techniques such as deep breathing exercises (not to be confused with inspiratory muscle training), meditation, yoga, guided imagery, and progressive muscle relaxation [16]. Although these interventions are well received by patients, a small randomized controlled trial by Haase et al. did not find that psychological interventions alone had a significant effect on pain intensity, analgesic consumption, or subjective postoperative fatigue [70]. In contrast, a meta-analysis by Tsimopolou et al. suggested positive effects of psychological prehabilitation on patient-reported outcomes [71].

Strictly speaking, psychosocial interventions must be separated from the psychological or psychiatric treatment of specific disorders. The reason for this is that assessing an individual’s response to therapy may require longer periods of time than those allowed in prehabilitation programs [72]. In consequence, studies assessing the effect of psychosocial prehabilitation should consider adjusting for these confounders. 

### 3.4. Functional

Inspiratory muscle training is the most common intervention of this group. In strict protocols, inspiratory muscle training consists of a series of inspiratory exercises with progressive increases in inspiratory resistance. The resistance is individualized to the patient’s baseline [73]. Other breathing exercises that can be considered functional training include incentive spirometry, pursed-lip breathing, and abdominal and thoracic breathing [74].

### 3.5. Cognitive

This intervention was recently introduced to specifically address the risk of delirium [75] but may be applicable to the broader concept of perioperative neurocognitive disorders [76]. Cognitive training includes the use of brain exercise games to improve memory, speed, attention, flexibility, and problem-solving. No baseline assessment, other than to rule out ongoing cognitive impairment, has been specified.

Special mention should be made on the case of neurosurgery, in which a delicate balance exists between maximal tumor eradication and the preservation of neurological function. Recently, the term neuromodulation-induced cortical prehabilitation (NICP) has emerged [77]. NICP refers to invasive (direct cortical stimulation) and noninvasive (e.g., transcranial magnetic or electric stimulation) neuromodulation coupled with intensive language or motor training [78]. Neuromodulation inhibits functional areas affected by the tumor, which are mapped with neurophysiological and neuroimaging [79,80]. By promoting neuroplasticity in distant areas, intensive training reduces the functional relevance of the tumor affected brain region, allowing more radical resections to improve survival. 

Overall, our review suggests that multimodal protocols are predominant in cancer populations. This finding is not surprising, as exercise has the potential to address multiple goals of prehabilitation with a single intervention (e.g., CRF, sarcopenia, nutrition). In multimodal and unimodal strategies, the most common intervention is exercise. Programs vary in the interventions, mode of delivery, schedules (e.g., number and duration of sessions), and goals. New prehabilitation interventions are being introduced, such as cognitive prehabilitation to address delirium and NICP for patients undergoing intracranial resections in anatomical regions of motor, language, or cognitive relevance.

## 4. How Is the Effect of a Prehabilitation Strategy Measured?

Randomized controlled trials in prehabilitation have been conducted for a wide variety of outcomes. Conceptually, these outcomes can be understood as clinical, patient-centered, or related to therapeutic effectiveness (Table 1). Therapeutic effectiveness outcomes have the advantage of proving that the intervention had the desired effect on the target function, either pre- or postoperatively [81]. For example, the effect of aerobic exercise can be estimated by calculating differences in CRF from baseline. For CRF, the gold standard is VO_2_max [47]. Surrogate measures of CRF may circumvent the practical limitations of CRF testing, including equipment, personnel, cost, and risk of side effects in patients with cardiovascular risk factors. The 6 min walking test (6MWT), for instance, has shown moderate correlation with VO_2_max in cancer patients [82]. It was developed as a variation of the 12 min walk test and has long been endorsed as a clinically useful and practical measure of submaximal functional capacity by the American Thoracic Society [83,84]. Expectedly, functional capacity (assessed by 6MWT) is the most common primary outcome in randomized controlled trials performed in adults undergoing thoracic surgery [55]. In a meta-analysis of five randomized controlled trials, Michael et al. found that exercise prehabilitation can improve the postoperative 6MWT in colorectal and esophagogastric cancer patients by an average of 58 m (95% CI: 23–92 m) [52]. Specifically for colorectal cancer, Falz et al. found that exercise improved the preoperative 6MWT by a mean of 30.8 m (95% CI: 13.3–48.3). For lung cancer patients, a multimodal (functional and aerobic) approach had a mean 6WMT improvement of 20 m (95% CI, 9.12–31.21) [74].

Improvements in muscle mass or strength from nutrition and resistance exercise in cancer patients have been mostly determined by the skeletal muscle index (SMI) and handgrip strength. The skeletal muscle index is a well-validated measurement that indexes the cross-sectional skeletal muscle area, ideally at the L3 vertebral level. Generally, sarcopenia is defined by an SMI below 34.4 cm^2^/m^2^ and 45.4 cm^2^/m^2^ in females and males, respectively [89]. Hand grip strength is measured with a hand dynamometer, most commonly the Jamar dynamometer. Results are reported in units such as Newtons, kilograms, or pounds per square inch [90]. To increase specificity, normalization by age, sex, and body height is also possible [91]. In a randomized controlled trial, Allen et al. demonstrated that prehabilitation ameliorated the preoperative decrease in skeletal muscle mass associated with neoadjuvant chemotherapy [60]. In a recent systematic review, Meneses-Echavez et al. reported that the minimum clinically important difference in strength is between 5 and 6.5 kg [14]. Regarding functional outcomes, the effect of inspiratory muscle training can be measured by inspiratory muscle pressures, forced vital capacity, and forced expiratory volume in 1 s [86].

Assessments of nutritional outcomes typically focus on changes in body composition, nutritional intake, functional capacity, clinical outcomes, and serum albumin levels. The Patient-Generated Subjective Global Assessment (PG-SGA) is widely used and validated self-reported questionnaire for the assessment of nutritional status in cancer patients [92]. The PG-SGA has a continuous scoring system to allow for prioritizing patients with more urgent needs, with a threshold score of 9 indicating a need for urgent nutritional intervention with 98% sensitivity for identifying malnutrition [93]. A systematic review by Novak et al. found that glutamine supplementation is associated with a reduction in infectious complication rates and shorter hospital stay without any adverse effect on mortality [94]. The greatest benefit was observed in patients receiving a high dose (higher than 0.20 g/kg/day) via parenteral administration. The least effective supplementation was a lower dose administered enterally to surgical patients. 

For the assessment of clinical outcomes, most studies have used a composite of postoperative complications, length of stay, and/or 30-day mortality. In the PREHAB trial, for example, Molenaar et al. randomized 269 patients undergoing colorectal cancer surgery to multimodal prehabilitation or standard of care (including ERAS). The co-primary outcomes were 6WMT and the composite of postoperative complications measured by the comprehensive complication index, which weighed complication incidence and severity into a single score [16]. Weighing complication severity is important and we consider it a better outcome than the simple rate of composite postoperative complications, which is prone to overestimate the importance of mild complications. In thoracic surgery, trials often focus on postoperative pulmonary complications. Pooled analyses of eight randomized controlled trials estimate that prehabilitation can decrease the incidence of pulmonary complications (OR 0.31, 95% CI: 0.2–0.48). Despite the effect size, the certainty of evidence was graded as moderate, with many trials indicating some or a high risk of therapeutic ineffectiveness [95]. In the PREPARE trial, the primary outcome was a validated score of postoperative pneumonia [96]. To ensure quality of outcome assessment, we recommend following the consensus definitions of postoperative pulmonary complications [97]. Length of stay and mortality outcomes are clinically meaningful and conceptually simpler outcomes, although they are more likely to be affected by factors other than the intervention itself. 

Examples of patient-centered outcomes include health-related quality of life and quality of recovery. Health-related quality of life can be estimated using questionnaire-derived scores, such as the short form health survey (SF-36) and the European Organization for Research and Treatment of Cancer quality of life questionnaire (EORTC QLQ-C30). The minimum clinically important differences are 5 points in the SF-36 and 4 to 11 points for improvement in the EORTC QLQ-C3 [14]. As for quality of recovery, the most common tools include the quality of recovery-40 and its abbreviated score, the quality of recovery-15 (QoR-15). Minimal clinically important differences for quality of recovery QoR-40 and QoR-15 are 6.3 and 8, respectively [88]. In a trial of 36 patients, Bojesen et al. demonstrated a mean QoR-15 score that was 21.9 points higher than in the control group [17]. A larger trial by Peng et al. detected changes in scores of life ability and physical well-being derived from the QoR-40 [98].

Few studies have addressed differences between unimodal or multimodal prehabilitation in cancer patients. Conceptually, multimodal programs are perceived better because they address more than one health dimension. In esophagogastric cancers, a subgroup analysis by Zhao et al. suggested that only multimodal prehabilitation was effective at reducing postoperative complications [99]. As most unimodal protocols consist of functional training, their results rather reflect the lack of therapeutic effectiveness of functional training and not on the modality of the program. 

In summary, prehabilitation can be evaluated by its therapeutic effectiveness or its effects on clinical or patient-reported outcomes. Therapeutic effectiveness is a necessary outcome when a specific intervention (including type, frequency, and duration) has not demonstrated the ability to reliably improve the targeted function. In turn, clinical and patient-reported outcomes are highly relevant because they constitute the principal basis for including prehabilitation interventions in future clinical practice recommendations. Studies should consider using definitions of minimum clinically important differences for sample size calculations and to demonstrate the relevance of statistically significant results, particularly in studies with small sample sizes. 

## 5. What Is the Suggested Effective Duration of a Prehabilitation Cancer Program?

The minimum effective duration of a prehabilitation program depends on the modality of the program and the population. In multimodal programs, the duration is determined (ideally) by the intervention that requires the longest time to produce a clinically relevant effect. In most cases, this intervention is exercise. To answer this question, one can consider existing evidence from sports medicine literature. Outpatient exercise programs can continue to see significant improvements for up to 36 weeks, but this duration far exceeds a reasonable preoperative waiting time. Falz et al. showed that studies in exercise prehabilitation for colorectal cancer reported consistent improvements in distance on 6MWT when the duration of the intervention was at least 3 weeks [100]. In contrast, the PREHAB trial, which used both aerobic and resistance exercise for 4 weeks, failed to show a significant difference in 6MWT, with a mean difference of 15.6 m (95% CI, −1.4–32.6; *p* = 0.07). In patients undergoing neoadjuvant therapy, longer interventions are feasible and have the potential to attenuate the negative effects of cancer therapy on functional status [60]. When considering exercise intensity, Huang et al. found that older sedentary adults can see improvements in VO_2_max with target heart rates as low as 35–50% of the heart rate reserve. Furthermore, training around 60% of the reserve is as effective as training at 70–80% [101].

When evaluating the impact of prehabilitation on complications, the results depend on how complications are interpreted. For instance, Falz et al. reported different (lower) rates of complications in the group of studies with longer prehabilitation programs, although the confidence intervals did not rule out the absence of a significant effect (>3 weeks duration: OR 0.66, 95% CI 0.4–1.1). Positive findings were also noted in a small randomized controlled trial of prehabilitation exercise for 3 weeks in colorectal surgery [102]. In PREHAB, which used a 4-week multimodal intervention including both aerobic and resistance training, the prehabilitation group had a lower rate of severe complications (17.1% vs. 29.7%; *p* = 0.02) as well as medical complications (15.4% vs. 27.3%, *p* = 0.02) but not in the overall incidence of all complications (31.7% vs. 42.2%, *p* = 0.07) or surgical complications (21.1% vs. 27.3%, *p* = 0.25). These findings underscore the need for discrimination between the types of complications, given that medical and surgical complications may be explained differently. 

For other prehabilitation interventions, evidence about the minimum effective duration is scarce. While follow-up is recommended for nutrition and psychosocial interventions, no specific interval assessment is recommended in guidelines for nutrition in cancer patients [92]. Unimodal functional prehabilitation for lung or esophageal cancer surgery has been used for shorter periods (1–2 weeks) [96,103]. It is important to note, however, that some interventions (e.g., nutrition, functional) benefit from exercise, and that psychosocial interventions may include qualitative, patient-reported benefits that exceed those measured by existing validated questionnaires and scores. Examples of these are the previously mentioned qualitative studies using patient interviews [53,104].

The ideal length of preoperative rehabilitation may conflict with surgical scheduling. Median waiting times are variable between surgeries, with Norway reporting waiting times close to 3 weeks for colorectal and breast cancers, shorter than that for lung or prostate cancer surgeries [105]. In pancreatic cancer, median waiting time was reported at 31 days but may extend beyond 56 days. Fortunately, waiting times alone do not appear to have a significant effect on survival for pancreatic [106], colorectal [107], gastric [108], and lung cancers [109].

In summary, the minimum effective duration for exercise is in most cases the intervention that determines the duration of a prehabilitation program. Without a high level of certainty, the existing evidence supports a minimum duration of 3 weeks. Significant uncertainty exists in the minimum effective duration for other forms of prehabilitation, but psychosocial interventions may provide qualitative benefits that are independent of the duration of such programs. 

## 6. Discussion

In this manuscript, we have performed an updated review of the rationale, interventions, outcomes, and minimal effective durations of unimodal and multimodal prehabilitation programs in adults undergoing cancer surgery. Perhaps the most consistent finding is that the heterogeneous methodology and the small sample sizes across studies preclude strong evidence-based recommendations for clinicians interested in implementing prehabilitation. Engel et al. found that the median number of participants in randomized controlled trials was 60 [10], similar to a median of 61 for the cancer-specific trials included by Meneses-Echavez [14]. The issue of small sample size is relevant to evaluate the risk of imprecision. The effect sizes are not only overestimated in the individual studies; they are also overestimated in pooled estimates [110].

Individual data from several large randomized controlled trials are now available. In colorectal cancer, PREHAB was an open-label, international, multicenter randomized controlled trial that assigned 251 patients to a comprehensive, center-based, multimodal prehabilitation program or standard of care. The trial was successful in demonstrating a decrease in severe complications, as well as medical complications, but could not confirm therapeutic effectiveness as determined by 6MWT or other subcomponent measures [16]. Of note, PREHAB had an estimated sample size of 714 but was stopped prematurely because the COVID-19 pandemic rendered it unfeasible. In contrast, the PHYSURG-C study tested a home-based multimodal protocol in a sample of 761 adults and found no difference in physical recovery at 4 weeks postoperatively [73].

In breast cancer, PHYSURG-B used a pragmatic approach of home-based, unsupervised physical activity (30 min daily for 2 weeks) on patient-reported measures of physical recovery among 316 women. While no significant differences were noted between groups, it is important to notice both had high rates of recovery (>80%). These results can help re-define priorities of prehabilitation in breast cancer. More recently, Machado et al. conducted PREPARE, a multicenter randomized controlled trial assessing the effect of home-based functional training on postoperative pneumonia in patients undergoing esophageal resection [96]. The trial randomized 241 patients to inspiratory muscle training or usual care and found small, yet statistically significant increases in inspiratory muscle strength and endurance. Such effects, however, did not decrease the risk of postoperative pneumonia (RR 1.1, 95% CI 0.79–1.53, *p* = 0.561). 

Cognitive prehabilitation has also been investigated. In the Neurobics trial, Humeidan et al. randomized 268 older adults undergoing major non-cardiac surgery to cognitive prehabilitation or usual care [75]. Enrollment commenced at least 8 days prior to surgery and consisted of cognitive training via an electronic tablet device. In patients with minimum compliance, the rates of delirium were 23% and 13.2% in the control and intervention groups, respectively (*p* = 0.04). After adjusting for surgical procedure and frailty, a lower risk of delirium was found in those undergoing cognitive prehabilitation (OR 0.58, 95% CI 0.33–0.99). The relatively wide confidence interval suggests that larger sample sizes will be necessary. 

Current literature supports the rationale of prehabilitation in cancer surgery. We present ample evidence that poor functional status, defined by functional capacity, nutritional status, etc., is associated with worse perioperative outcomes. In fact, unfit adults are to benefit the most from physical activity and are more likely to show significant increases in functional capacity [111]. Moreover, the existing evidence provides reasonable guidance to test and measure therapeutic effectiveness but is yet to confirm which protocol yields better results. To prioritize future research efforts, we refer readers to a recently published international consensus specific to patients undergoing cancer surgery [112].

We acknowledge some limitations in our review. First, the broad scope of this topic limits our ability to include all existing sources in a single manuscript. Systematic reviews and meta-analyses may be best suited to answer specific clinical or scientific questions. Second, prehabilitation may imply additional costs to patients and healthcare systems, but assessment of cost effectiveness was considered out of the scope of this review. Third, we notice that the literature in prehabilitation is growing at a rapid pace. Our current understanding is thus subject to change dramatically.

## 7. Conclusions

Cancer surgery places a significant burden on patients’ functional status and quality of life. Adults undergoing cancer surgery stand to benefit from effective prehabilitation programs. As of now, prehabilitation has demonstrated the potential to modify the risk of postoperative complications and improve patient-reported measures including quality of life and quality of recovery after surgery. Unfortunately, the level of certainty from multiple systematic reviews and meta-analyses is still suboptimal. More recent high-quality trials, which included larger sample sizes and better methodology, still show conflicting results. Due to large methodological heterogeneity, future trials should continue to use measures of therapeutic effectiveness so that more specific prehabilitation protocols can be studied and recommended. The results of ongoing clinical trials may shape our understanding of the benefits of prehabilitation in cancer patients [80,113,114,115].

## Figures and Tables

**Table 1:** Common outcomes measured in randomized controlled trials of prehabilitation in cancer surgery.

Outcome	Type of Outcome	Applicable Intervention	What it Measures	Minimal Clinically Important Difference
Maximal Aerobic Capacity	Therapeutic Effectiveness	Exercise (Aerobic)	Oxygen consumption at maximal effort (~80–85% age-adjusted predicted heart rate)	1–2 METs * [47]
6 min walking test	Therapeutic Effectiveness	Exercise (Aerobic)	Distance (m) at submaximal aerobic capacity	Not standardized, 70 m ^+^ [83]
Hand Grip Test	Therapeutic Effectiveness	Exercise (Resistance)	Muscle strength (Kg, N, etc.)	5–6.5 Kg [14]
1 RM chest press	Therapeutic Effectiveness	Exercise (Resistance)	Muscle strength (Kg)	Not reported
Patient-Generated Subjective Global Assessment (PG-SGA)	Therapeutic Effectiveness	Nutrition	Global numerical nutritional assessment	9 points ^‡^ [85]
Albumin	Therapeutic Effectiveness	Nutrition	Serum albumin concentration (g/dL)	Normal values 3.5–5.5 d/dL
Maximal Inspiratory Pressure	Therapeutic Effectiveness	Functional	Improvement of IMT	Not reported [86]
Rate of Postoperative Complications	Clinical	Non-specific	Proportion (%)	Not standardized
Comprehensive Complication Index	Clinical	Non-specific	Severity-weighed complication rate 0 (no complication)–100 (death)	CCI > 20 indicates severe complications [16]
Length of Stay	Clinical	Non-specific	Hospital stay in days	NA
Mortality	Clinical	Non-specific	Proportion of deaths at different time points	NA
PHQ-9	Patient Health Questionnaire	Psychological	Questionnaire-based score for depression	3.7 points [87]
GAD 7-item	Generalized Anxiety Disorder	Psychological	Questionnaire-based score for anxiety	3.3 points [87]
SF-36	Patient-centered	Any intervention	Health-related quality of life	5 points [14]
EORTC QLQ-C30	Patient-centered	Any intervention	Health-related quality of life	4–11 points [14]
Quality of Recovery 40	Patient-centered	Any intervention	Multidomain measure of postoperative recovery	6.3 points [88]
Quality of Recovery 15	Patient-centered	Any intervention	Abbreviated multidomain measure of postoperative recovery	8 points [88]

EORTC QLQ-C30: European Organization for Research and Treatment of Cancer quality of life questionnaire. GAD: generalized anxiety disorder. IMT: inspiratory muscle training. NA: not applicable. PG-SGA: Patient-Generated Subjective Global Assessment. PHQ-9: Patient Health Questionnaire 9-item. SF-36: short form health survey. *: Based on long-term general population studies. ^+^: 9 points are reported to change nutritional stage class in an ordinal nutritional scale (SGA). ^‡^ 95% confidence that the improvement is clinically significant.

## Data Availability

Publicly available publications referenced in the submission and listed below were analyzed in this review.
